# Somatic multicellularity as a satisficing solution to the prediction-error minimization problem

**DOI:** 10.1080/19420889.2019.1643666

**Published:** 2019-07-28

**Authors:** Chris Fields, Michael Levin

**Affiliations:** a Caunes Minervois, France; b Allen Discovery Center at Tufts University, Medford, MA USA

**Keywords:** Ancestral genetic toolkit, cellular information processing, free-energy principle, markov blanket, percolation theory, ur-metazoan

## Abstract

Adaptive success in the biosphere requires the dynamic ability to adjust physiological, transcriptional, and behavioral responses to environmental conditions. From chemical networks to organisms to whole communities, biological entities at all levels of organization seek to optimize their predictive power. Here, we argue that this fundamental drive provides a novel perspective on the origin of multicellularity. One way for unicellular organisms to minimize surprise with respect to external inputs is to be surrounded by reproductively-disabled, i.e. somatic copies of themselves – highly predictable agents which in effect reduce uncertainty in their microenvironments. We show that the transition to multicellularity can be modeled as a phase transition driven by environmental threats. We present modeling results showing how multicellular bodies can arise if non-reproductive somatic cells protect their reproductive parents from environmental lethality. We discuss how a somatic body can be interpreted as a Markov blanket around one or more reproductive cells, and how the transition to somatic multicellularity can be represented as a transition from exposure of reproductive cells to a high-uncertainty environment to their protection from environmental uncertainty by this Markov blanket. This is, effectively, a transition by the Markov blanket from transparency to opacity for the variational free energy of the environment. We suggest that the ability to arrest the cell cycle of daughter cells and redirect their resource utilization from division to environmental threat amelioration is the key innovation of obligate multicellular eukaryotes, that the nervous system evolved to exercise this control over long distances, and that cancer is an escape by somatic cells from the control of reproductive cells. Our quantitative model illustrates the evolutionary dynamics of this system, provides a novel hypothesis for the origin of multicellular animal bodies, and suggests a fundamental link between the architectures of complex organisms and information processing in proto-cognitive cellular agents.

## Introduction

The unicellular lifestyle dates back to the origin of life on Earth some 3,900 million years ago (Mya) [1] and remains successful today, with the vast majority of genetic as well as niche diversity found in the unicellular microbial world [2]. Unicellular prokaryotes and later, eukaryotes also developed facultatively multicellular lifestyles, e.g. in microbial mat communities, involving mutual dependence and division of labor (reviewed by [3–5]). Eukaryotes developed, in addition, the obligate multicellular lifestyle; indeed the eukaryotic cell is itself sometimes regarded as the initial obligate multicellular organism [6]. Unlike facultative multicellularity, obligate multicellularity compromises or altogether eliminates the independent reproductive fitness of at least some of the cells involved. What would induce cells to give up independent reproductive fitness in favor of a completely-dependent lifestyle as part of an obligate multicellular organism? This question is often answered by the analogy of a “contract” in which cells cooperatively surrender independent reproductive fitness for the benefits of group selection [7,8]; however, the mechanisms driving such arrangements have remained unclear. Here we suggest that obligate multicellularity provides a solution, typically satisficing not optimizing, to the problem of minimizing errors in the prediction of the behavior of the environment. The selection pressure for obligate multicellularity is, if this is correct, essentially thermodynamic [9,10].

While facultative multicellularity is commonplace among both prokaryotes and eukaryotes, obligate morphologically-complex multicellularity is rare, occurring in only five eukaryotic clades (reviewed by [1,11,12]). Somatic multicellularity, in which cell division is restricted wholly or significantly to specialized stem and/or germ cells, is characteristic of the Metazoa. Following Haeckel [13], it has long been theorized that ancestral metazoa were clonal colonies of morphologically-identical cells. Recent ultrastructural and phylogenomic studies have established choanoflagellates as the closest extant unicellular relatives of metazoa [14] and provided significant new evidence to support James-Clark’s [15] hypothesis that choanoflagellates are homologous to sponge choanocytes, and hence that facultative rosette colonies of choanoflagellates are plausible models of ancestral multicellularity [16]. Such studies also suggest that multicellularity may be a Holozoan character predating the choanoflagellate – metazoan split [1,12]. Funayama [17,18] has suggested that choanocytes are ancestral to the ameboid archeocyte stem cells of demosponges, consistent with the observation of alternating flagellate and ameboid forms elsewhere in the unicellular holozoa [12].

Obligate, morphologically-complex multicellularity is a canonical major transition in evolution, establishing the multicellular organism as a Darwinian individual characterized by genetic interests and reproductive fitness [6,19]. As Szathmáry [6] emphasizes, the division of labor possible with multicellularity enables fitness-increasing synergistic interactions between distinct cell types, and hence specific selection for such interactions. Models of the evolution of complex multicellularity have, accordingly, focused on fitness and selection at the whole-organism level [7,8,20–22]. Motivated by Solana’s [23] primordial stem cell hypothesis, experimental and modeling results suggesting that stem cells (neoblasts) in asexual freshwater planaria remain Darwinian individuals [24] and by a conception of cancer as an escape from organism-scale control [25–28], here we consider the selection pressures that could drive a transition to multicellularity from the perspective of a single reproductive cell. We suggest that the largely-somatic multicellular bodies of metazoa can, in general, be considered protective environmental niches constructed by stem and/or germ cells to assure their own future reproductive fitness. We formulate this suggestion in terms of the Free Energy Principle (FEP) defined by Friston [9,10] as the claim that reproductive (proto-)stem cells construct multicellular bodies in order to reduce the variational free energy of information exchange with the immediate environment and hence to minimize prediction error as regards their own survival. From this FEP perspective, somatic multicellularity is a natural outcome of environmental challenges faced by free-living cells, with the primary genetic driver of multicellularity being the development of intercellular signals for cell-cycle arrest. In this “imperial” model of multicellularity [24], reproductively-competent stem and/or germ cells produce reproductively-incompetent somatic cells in order to advance their own genetic interests by establishing an environment-facing cellular layer that reduces uncertainty and improves predictive power (and thus physiological adaptation) for the internal cells.

We begin by considering the effects of increasing environmental lethality, due to predators, pathogens, toxins or other threats, on populations of dividing cells in environments with different resource levels. Using a simple parametric model, we show that the combination of high resource levels with high lethality favors a phase transition from a rapidly-dividing population to one in which significant resources are devoted to reducing environmental threats. We then generalize from environmental lethality to any source of high variational free energy [9,10]. We suggest that a protective environment composed of reproductively-disabled progeny provides a satisficing, though not necessarily optimal, solution to the problem of minimizing variational free energy and hence prediction error. Using a quantitative model of stem and somatic cells in an external environment characterized by varying levels of lethality, we investigate the parameter dependence of the transition to multicellularity. We show that a protective multicellular body can be modeled as a Markov blanket that undergoes a phase transition to informational opacity as the environmental free-energy buffering capacity of somatic progeny is increased. Finally, we review evidence that the ancestral genetic toolkit of holozoans is sufficient to support the transition to multicellularity. We discuss these results from two perspectives: that of individual cells as information processing units that can be assembled into a computational interface with the environment, and that of cancer as an escape by somatic cells from “imperial” oversight and control.

## Methods

The model simulation employs a 30 by 60 grid of possible cell locations (1800 total) with lethality varying from 0% per stem cell division cycle to 100% per stem cell division cycle along the long axis in 60 equal increments. Stem cells are seeded in random positions at 5% density. The probability that a stem cell will die before reproducing is determined by the lethality *b* of the cell it occupies, multiplied by the protection β it receives from neighboring somatic, i.e. non-reproductive cells, if any.

The level *a* of reproductive resources for stem cells in the environment is fixed by a parameter setting. The local resource level α*a*, where the parameter α represents the availability of reproductive resources to a stem cell at a given location, determines the probability of stem-cell division at that location on each cycle. The value of α for a stem cell is reduced by 20% for each neighboring stem cell by default; this reduction parameter is adjustable. If the production of somatic progeny is enabled, the resource availability α for a stem cell is increased by 20% for each neighboring somatic cell by default; this parameter is also adjustable.

Stem cells are only allowed to divide if they have a neighboring open cell; this restriction models the relative rigidity of the somatic body.

Somatic cells protect neighboring cells, stem or somatic, by an adjustable factor β. Somatic cells resist lethality by an adjustable factor, with a default of 20%.

The model simulation is implemented in javascript using the HTML 5 Canvas function for display. All probabilities are calculated by comparing random numbers generated by the javascript Math.random function to parameterized limit values. The simulation is available for use and its source code can be examined at https://chrisfieldsresearch.com/somatic-protection-model.htm.

## Results

### Environmental challenges drive a phase transition from reproduction to protection

A multicellular body composed primarily of reproductively-disabled somatic cells is a eukaryotic innovation widely recognized as a major evolutionary transition [6,19]. Why, however, would an ancestral eukaryotic cell devote reproductive resources to the generation of non-reproductive progeny? Doing so manifestly decreases reproductive fitness. What selective environment would tolerate and even reward this fitness-decreasing strategy long enough to enable the cellular differentiation and synergistic interactions characteristic of complex multicellularity to arise?

Here we suggest that a high-resource environment that is also high in potential lethality, e.g. due to the presence of predators, pathogens, toxins, changing conditions, or other threats, rewards the investment of reproductive resources to produce a multicellular somatic body, provided the body is capable of protecting its reproductive cells from the environment. Such a high-lethality environment imposes a conflict between the demands of short-term reproductive fitness and short-term survival; a protective body resolves this conflict in favor of survival at the expense of fitness. In this picture, the primary evolutionary innovation enabling the multicellular lifestyle is not increased cooperation between reproductively-competent cells as suggested by either “fraternal” or “egalitarian” aggregation-based models [7,8] (see also [29] for a review of aggregation-based models), but rather the ability of a reproductively-competent cell to actively suppress the reproductive potential of its somatic progeny. This innovation is most fully expressed in obligately-sexual organisms in which a single, gamete-producing stem cell lineage, i.e. a specific germ lineage, is the sole carrier of reproductive fitness.

To describe this situation quantitatively, consider a uniform population of free-living, reproductive cells in a resource-rich environment, and let τ be the minimal cell-cycle time for these cells. If every cell divides in each period of length τ, then the population size *N*(*t*) after *n* cycles, i.e. at *t* = *n*τ is:

$$N\left(t\right)=N_{0}\cdot 2^{n}$$

where *N*_0_ is the initial population size. If only *r* of the cells divide on each cycle, we can write:

$$N\left(t\right)=N_{0}\cdot r^{n}$$

Now let us consider an environment in which:

$$r=\left(1+\alpha a\right)\cdot \left(1-\beta b\right)$$

where 0 ≤ *a,b* ≤ 1 are parameters describing the environment and 0 ≤ α,β ≤ 1 are parameters describing the cells. These parameters are interpreted as follows:

- *a* measures the availability of resources for reproduction in the environment, with *a* = 0 being starvation conditions allowing population maintenance only and *a* = 1 being sufficient resources for (in practice) unlimited growth.
- *b* measures the lethality of the environment, with *b* = 0 being completely benign and *b* = 1 being complete lethality for the population in question.
- α measures the efficiency with which available resources are employed for reproduction by a given cell, with α = 0 being minimal and α = 1 maximal efficiency.
- β measures the degree to which dividing cells are exposed to the environment, with β = 0 complete protection from the environment and β = 1 complete exposure.

Maximum population growth is clearly achieved when *a* = α = 1 and either *b* = 0 or β = 0.

**Figure 1.**
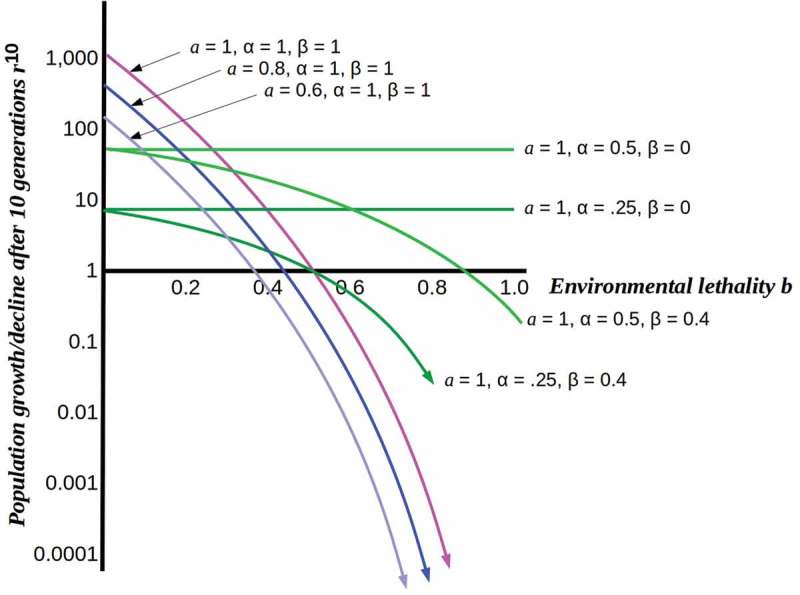
Plots of population growth *r^n^* for *n* = 10 from a single initial cell as functions of environmental lethality *b* under different assumptions. Pink, blue and purple curves show the effect of decreasing resource levels on the rate of population collapse as lethality increases. Light and dark green curves show relative stability of fully (β = 0) or partially (β = 0.4) protected populations at different levels of resource-use efficiency.

For independent, free-living cells, we can set α = β = 1, i.e. the cells employ all available resources for reproduction and are fully exposed to the environment. In this case, increasing the environmental lethality *b* causes population collapse as shown in Figure 1, with populations in resource-poor environments (i.e. *a* < 1) collapsing sooner but no populations able to maintain growth above *b* = 0.5.

What happens, however, when cells are able to divert some fraction of the available resources from reproduction to protection, i.e. to shielding themselves from the environment? At high resource levels and low lethality, this is a low-fitness strategy: the resulting protected populations are lower than unprotected free-living populations, even when losses due to environment lethality are taken into account. As lethality increases, however, this ceases to be the case, as shown in Figure 1. Population survival past *b* = 0.5, in particular, requires protection from the environment regardless of resource level or resource-usage efficiency. A fitness-optimizing population would, therefore, be expected to undergo a phase transition from unprotected to protected at a critical point in *b*, the lethality level at which the declining free-living population reached the protected population sustainable at the achievable values of α and β. Population survival (i.e. *r* ≥ 1) requires βb ≤ α*a*/(1 + α*a*). We show below that this phase transition can be represented as crossing a percolation threshold, here given by the above inequality.

The phase transition shown in Figure 1 is, we suggest, the phase transition to complex multicellularity implemented by the free-living holozoan ancestors of the metazoa. It is highly plausible that the period between the rise of heterotrophic eukayotes, i.e. predators roughly 800 Mya [1] and the appearance of multicellular Ediacaran fauna roughly 575 Mya saw not only rapid eukaryotic diversification but multiple instances of population-scale exposure to high-lethality environmental change. Such changes can be expected to have imposed severe selective pressure inducing multiple population bottlenecks. In the sections that follow, we employ Friston’s [9,10] FEP to develop a scenario in which a reproductively-disabled somatic body is a solution to the challenge of protecting oneself from a challenging environment.

### Survival as minimization of prediction error

The FEP characterizes organisms as systems that behave so as to minimize the variational free energy (VFE) of their environments [9,10]. The VFE of the environment measures the unpredictability – the “variational freedom” – of the environment and hence the mismatch between the environment’s behavior and the organism’s expectations about its behavior. Zero VFE corresponds to perfect predictability of its environment by an organism or, equivalently, of an organism by its environment. Minimizing net information flow minimizes net energy transfer; hence minimization of VFE is a least-action principle analogous to the energetic least-action principles of physics [30]. In a Bayesian setting, zero VFE corresponds to equality between posterior and prior probabilities, where both provide completely accurate accounts of outcomes. While the FEP has primarily been applied in a cognitive-neuroscience context, it is a general principle applicable to organism-environment dynamics at evolutionary, developmental, or learning time scales [10,31].

Living beings face numerous challenges due to the ever-changing chemical, physical, and biological events in their immediate environment. Successful organisms, tissues (e.g. populations of neurons [32]), and even societies (e.g. swarm intelligence [33]) must act in order to adaptively respond to these challenges [34,35]. Gene-regulatory networks, organelle machinery, cell behaviors, system-level physiological changes, and behavior are among the mechanistic options available to biosystems at multiple scales in the face of stresses. As Friston [10] has emphasized, maintenance of homeostasis is the fundamental predictive problem faced by any organism, and the probability of continuing homeostasis is the fundamental prior probability for any organism. To incorporate reproductive fitness, we can extend the meaning of “homeostasis” to include maintaining the cell cycle at the rate allowed by the environment. Prediction failures, i.e. differences between posterior and prior probabilities of continuing homeostasis, including continuing reproduction, can be addressed either by revising priors (i.e. learning) or acting in a way that alters the posteriors (“active inference”). An important special case of the former is revision of Bayesian precision assignments to either environmental inputs or expectations, implemented in humans by changes in salience and attention [36].

A threatening environment challenges the maintenance of homeostasis and therefore has high VFE; a lethal environment takes this challenge to the extreme. We can, from this perspective, see the phase transition from growth to protection shown in Figure 1 as an instance of active inference, i.e. behavior change that preserves the prior probability for continuing homeostasis. What is this behavior change? Merely curtailing cell division is not by itself protective. In facultative multicellular eukaryotes such as *Dictyostelium*, mitotic arrest followed by aggregation, collective motility, differentiation and spore formation increases the probability of population-level survival. The *Dictyostelium* spore, like spores in general, is a differentiated form that isolates the cellular components needed for later reproduction from the environment. Spore-generating cells actively induce the cell-cycle arrest and differentiation of the supporting stalk cells, which do not contribute DNA to the subsequent population, by secreting small-molecule morphogens [37,38]. Here we suggest that obligate multicellular organisms adopt a very similar strategy, in which reproductive cells actively induce cell-cycle arrest and differentiation by cells dedicated to protection against the environment. The key difference between obligate and facultative multicellular strategies is that in obligate multicellular organisms, reproductive cells induce differentiation and disable reproduction in their own progeny.

To make this suggestion precise, consider a cell **C** with time-varying state **c**(*t*) is embedded in an uncertain environment **E** with time-varying state **e**(*t*). Consider the cellular state **c**(*t*) to have two orthogonal components, **c**(*t*) = **a**(*t*) × **p**(*t*) defined by the the actions of functions:

$$A:E\to C,A:e\left(t\right)\to a\left(t+\delta t\right)\;and\;P:C\to C,P:c\left(t\right)\to p\left(t+\delta t\right),$$

for some suitably small time increment δ*t*. We interpret ***A*** as the *action* of **E** on **C** to produce an “input state” **a**(*t*+ δ*t*) and ***P*** as the *prediction* by the cell **C** of its future “internal state” **p**(*t*+ δ*t*). The implementations of these state components may be, for example, the state of all membrane-bound receptors and channels at *t* for **a**(*t*) and the combined metabolome and transcriptome state at *t* for **p**(*t*). The prediction implemented by ***P*** is accurate (optimal) if the cell’s behavior given **p**(*t*) is an optimal response to the environmental conditions **e**(*t*) and hence to **a**(*t*); in this case, we can (abusing the notation somewhat) write **p**(*t*) = **a**(*t*). The prediction is satisficing if **C** is able to maintain homeostasis; clearly some satisficing solutions have higher fitness than others. The prediction function ***P*** implements, in this case, a “model” of the **E** – **C** interaction, in the sense defined by Conant and Ashby [39] in stating the good-regulator theorem; **C** can be viewed as regulating its interaction with **E** by using this model. Having an accurate model minimizes prediction error and maximizes fitness within the constraints dictated by the environment.

**Figure 2.**
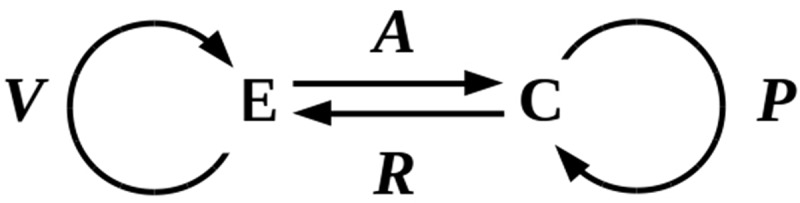
Interaction between an environment **E** and an embedded cell **C**. The functions ***V*** and ***P*** specify environmental variation and cellular predictions, respectively.

We can, similarly, consider the environmental state **e**(*t*) to have two orthogonal components, **e**(*t*) = **r**(*t*) × **v**(*t*) defined by the the actions of functions:

$$R:C\to E,R:c\left(t\right)\to r\left(t+\delta t\right)\;and\;V:E\to E,\;V:e\left(t\right)\to v\left(t+\delta t\right),$$

where we interpret ***R*** is the *reaction* of **C** on **E** and ***V*** is the *variation* by **E** that generates its future state. From an informational perspective, the components **C** and **E** are symmetric; however, for simplicity we can assume **E** is “large” enough that ***R*** is negligible, so that **e**(*t*) ~ **v**(*t*). The interaction between **C** and **E** is summarized in Figure 2. The prediction problem faced by **C** is the problem of minimizing the difference Δ(*t*) = |**a**(*t*) – **p**(*t*)| between its input **a**(*t*) from **E** and its prediction **p**(*t*). An accurate or optimal solution to this minimization problem has Δ(*t*) = 0; a satisficing solution has 0 < Δ(*t*) ≤ Δ_lethal_, where Δ_lethal_ is the prediction error sufficient to cause cell death.

As a simple example, suppose the states **c**(*t*) are vectors in **R**^2^ with norms |**c**(*t*)| ≤ 1 for all *t*, the states **e**(*t*) are real numbers in [0,1], the functions ***V, A, R, P*** [0,1]: → [0,1], the value ***V***(**e**(*t*)) and hence **e**(*t*) is random as a function of *t*, and the function ***A***(*t*) can be represented by **a**(*t* + 1) = β* ·* **e**(*t*), with 0 ≤ β ≤ 1 as above representing the degree to which **a**(*t*) is “exposed” to **e**(*t*). Assume that ***P*** is the “homeostatic success” prediction that **E** and ***A*** are invariant, i.e. **p**(*t* + 1) = **a**(*t*). In this case, the prediction error Δ → 0 as β → 0. As β → 1, Δ(*t*) → random due to the randomness of **e**(*t*). The lethality *b* of the environment can, in this case, be identified with the level Δ_lethal_ of prediction error that is fatal; the fraction of cells that implement insufficiently accurate predictions and therefore die is β*b* = βΔ_lethal_.

This model can clearly be extended to *d* real dimensions, variation in each of which is restricted to [0,1], representing distinct degrees of freedom of **E** that **C** needs to predict sufficiently accurately to maintain homeostasis and hence survive. If these dimensions are independent (i.e. orthogonal in R^2*d*^) and a prediction failure for any one dimension *k* is lethal (i.e. Δ_*k*_ > Δ_lethal_(*k*)), then any one of 2*^d^*^−1^ distinct prediction failures will be lethal. In this case, survival is reasonably probable only if the environmental exposure β ~ 2^−*d*^. Free-living cells with *d* independently-lethal dimensions thus face an exponential prediction problem; solving this problem and hence surviving is clearly infeasible as *d* becomes large. Controlling the effective dimensionality of the environment is thus a key component of VFE reduction.^1^ Geometrically-symmetrical aggregates of identical cells in which all cells are equally exposed to the environment, e.g. *Volvox* colonies or choanoflagellate rosettes, provide protection against unicellular predators [40], but do not decrease β along other dimensions. Metazoan embryos that take this form are either protected within a specialized environment – an egg or womb – or suffer high mortality.

### Minimizing prediction error with reproductively-disabled progeny

**Figure 3.**
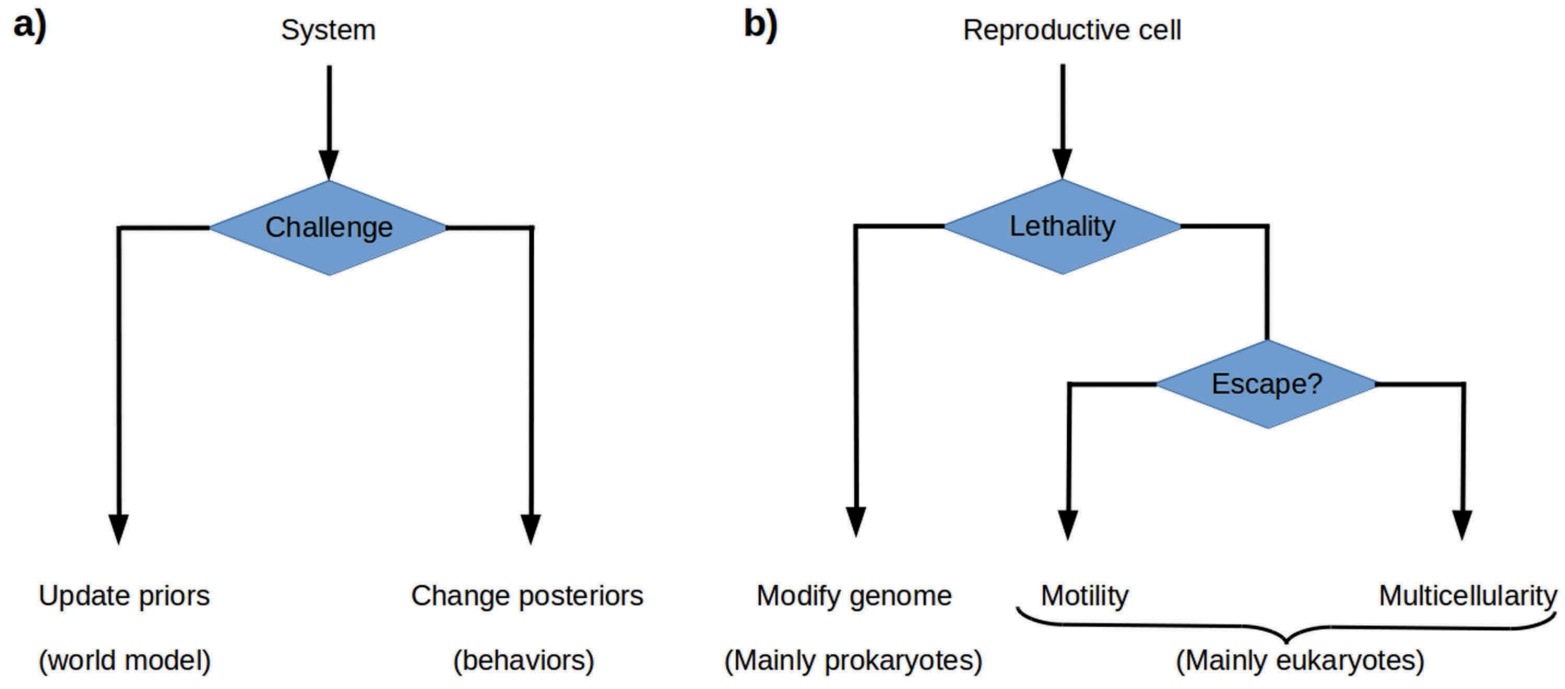
a) The theory of active inference postulates that systems challenged by high environmental free energy respond either by updating their prior probability distributions or world models, or by altering their posterior probability distributions by acting on the world so as to change its effects on them. b) Reproductive cells challenged by environmental lethality can be expected either to update their prior probability distributions by altering their genomes, or to execute some behavior that changes their environment’s lethality. Genome modification by rapid evolution or lateral gene transfer is primarily a prokaryotic strategy. Motility is a successful, primarily eukaryotic strategy when environmental lethality varies over short ranges. Building a multicellular structure, a body, that protects reproductive cells from the environment provides a solution when escape is not possible.

From an FEP perspective, one would expect cells to respond to increasing environmental lethality in one of the two ways given by the theory of active inference: either by changing their prior probability distributions and hence their regulatory models or by acting so as to change their environments and hence their posterior, i.e. input, probability distributions.^2^ Modifying the genome is the most available way of altering the prior probability distribution, i.e. the generative model of the environment, for prokaryotes; the enormous genetic diversity of prokayrotes [2,41] suggests that genome modification is the primary prokaryotic strategy for dealing with environmental change. Rapid genome modification by lateral gene transfer is ubiquitous among prokaryotes, with environmental-response genes particularly frequently transferred [42–44]. Motility is the most available way of altering the posterior probability distribution; the eukaryotic development of the cytoskeleton enabled multiple motility solutions for unicellular eukaryotes, with environment-dependent morphological changes supporting different motility solutions, e.g. flagellar propulsion and ameboid crawling, available in many lineages [12]. Motility is, however, only useful if the lethality of the environment varies significantly over short distances and hence permits escape. Somatic multicellularity can, we suggest, be understood as a more radical solution to the problem of altering the posterior probability distribution in an environment in which lethality varies only over longer ranges (Figure 3). The reproductive cells within an obligate multicellular organism alter their environment by constructing and then inhabiting a new environment that they largely control, i.e. one with consistently low VFE. The components employed for this construction project are their own progeny, the most predictable living components available.

The most important criterion for a synthetic, protective environment is that it not be or become a competitor. From a fitness perspective, the simplest way to assure non-competition is by constructing the environment out of cells or other materials, e.g. extracellular matrix components, that have no independent genetic interests or reproductive fitness. Such materials cannot respond to environmental variation by varying their allocation of resources to reproduction; hence they are intrinsically more predictable than reproductively-competent cells. Hence a system – a reproductive cell – driven to minimize VFE can be expected to build a somatic body comprising non-reproductive cells as a protective environment in preference to either a clonal (i.e. fraternal) or aggregative (i.e. egalitarian) body composed of other reproductively-competent cells. The demosponges, with a single class of proliferative, totipotent stem cells (the archeocytes), exemplify this body-building strategy [17], suggesting that it may be ancestral in the Metazoa. That a single class of proliferative, totipotent stem cells (the neoblasts) are also found in planaria [24] indicates that an all-or-nothing strategy for regulating cellular reproduction was maintained at least into early-branching bilaterals.

**Figure 4.**
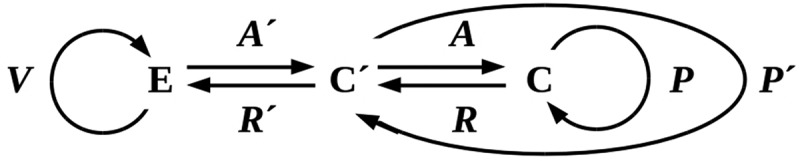
Adding a buffer between **C** and **E** by reproducing a copy **C’** of **C**. Competition for reproduction-enabling resources is eliminated **C’** if reproductively disabled.

To extend our earlier model to this situation, suppose that **C** can reproduce a copy **C’** of itself with time-varying state **c’**(*t*) = **a’**(*t*) × **p’**(*t*) as above, and interpose this **C’** as a protective buffer between itself and **E** as shown in Figure 4. Protection by **C’** replaces the exposure β of **C** with some β_prot_ < β; if protection is complete, β_prot_ = 0. Assume as above that the reactions ***R*** and ***R’*** are negligible, and consider the simplest case, reproduction of an exact copy **C’** with prediction mapping ***P’* **=** *P*** and environmental exposure β**’** = β. In this case, the probability of **C’** dying and requiring replacement to prevent re-exposure of **C** to **E** in any reproductive cycle is β**’** Δ_lethal_; if β**’** Δ_lethal_ ≥ 0.5α*a*, all available resources must be dedicated to reproduction for protection, and **C** remains exposed to **E** during part of each cell cycle. Hence the ability of **C** to maintain **C’** is critically dependent on the availability of reproductive resources and their efficient use by **C**. As before, population survival is only possible if β**’**Δ_lethal_ ≤ α*a*/(1 + α*a*). Reproducing an exact, reproductively-competent copy as protection against a lethal environment is, therefore, no better as a strategy than aggregation.

Effective protection from a lethal environment requires the production of progeny or materials that have decreased environmental sensitivity relative to **C**, i.e. that have β**’** < β. Non-reproductive cells can be expected to have β**’** < β, due both to their lower metabolic requirements [45,46] (see also below) and their ability to re-tool metabolism toward the production of either inert protective materials or active defenses. The production of long-lived non-reproductive progeny increases fitness if it allows the subsequent production of protected reproductively-competent progeny, e.g. daughter stem cells. If *k* protective cells must be produced every *m* cell cycles, with *k* < *m*, the population of reproductive cells is able to grow as r^(*n*/*m*)(*m* – *k*)^ over an elapsed time *n*τ; for *k* ≪ *m*, this approaches the best-case rate *r^n^*.

**Figure 5.**
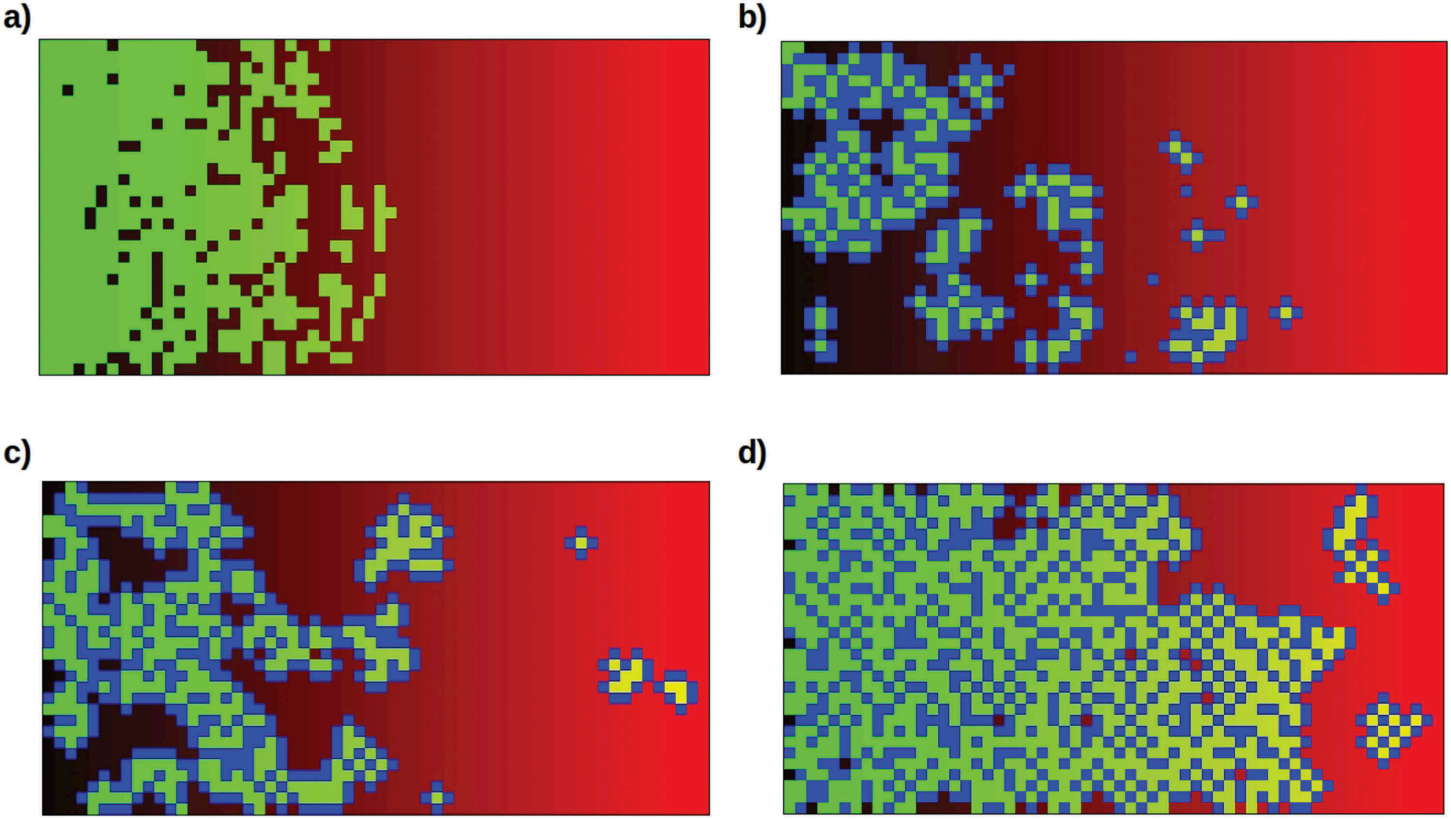
Typical model simulation runs under different conditions. The simulation depicts an environment varying in lethality from 0% (*b* = 0, black, left margin) to 100% (*b* = 1.0, red, right margin). Stem cells (green) are randomly seeded in this environment at a density of 5% and allowed to reproduce for 120 division cycles. Neighboring stem cells compete for reproductive resources. Somatic progeny (blue) protect their nearest-neighboring cells, whether these are stem or other somatic cells. a) Stem cells alone cannot invade the higher-lethality half of the environment, even with optimal reproductive resources (*a* = 1.0). Here neighboring stem cells suffer a 20% resource reduction (α = 0.8) due to competition; removing this reduction does not allow them to cross the 50% lethality boundary. b) Moderately (50%, i.e. β**’** = 0.5) protective but relatively long-lived (50% resistance to lethality) somatic cells allow mainly small colonies in the moderately-lethal (50% – 75%) sector of the environment (*a* = 0.8, probability of somatic-cell progeny 50%). c) Fully (100%, β**’** = 0) protective but shorter-lived (only 20% resistance to lethality) somatic cells allow larger colonies to populate even the high-lethality (75% – 100%) sector of the environment (*a* = 0.8, probability of somatic-cell progeny 50%). d) Fully-protective and relatively long-lived (50% resistance to lethality) somatic cells allow invasion of the lethal sector of the environment (*a* = 1.0, probability of somatic-cell progeny 25%).

To investigate this model of non-reproductive, i.e. somatic progeny as highly-predictable protectors of reproductive cells, assumed here to be totipotent stem cells as in demosponges or planaria, from the external environment, we have developed a quantitative simulation that embeds stem cells in an environment of varying lethality and allows parameters governing their ability to reproduce either stem or somatic progeny to be manipulated (see **Methods**). Consistent with the model, unprotected stem cells cannot successfully invade an environment with greater than 50% lethality (*b* = 0.5) on each division cycle even if reproductive resources are optimal (Figure 5(a)).

**Figure 6.**
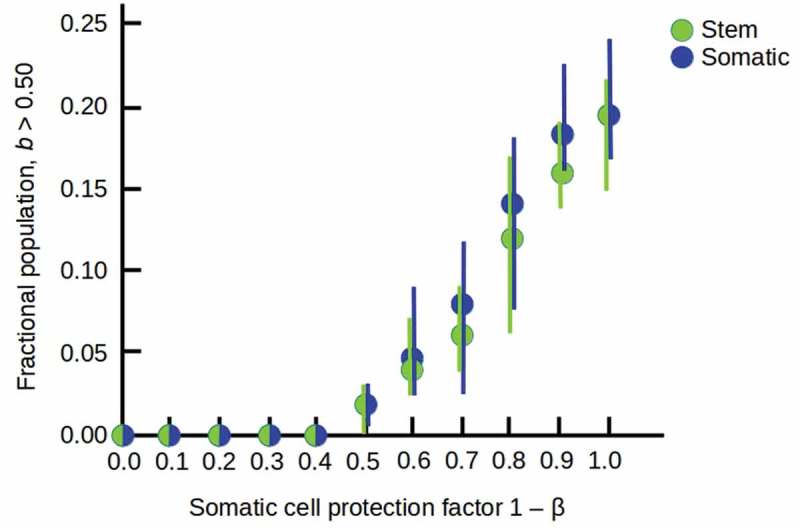
Increasing occupation of the high-lethality (*b* > 0.5) half of the environment as the somatic cell protection factor 1 – β is increased from 0.0 to 1.0, with all other parameters as in Figure 5c. Filled circles show averages over 10 runs with random starting populations; bars show full range of results obtained.

When the production of somatic progeny is enabled, different combinations of protective ability and resistance to lethality lead to different abilities for stem cells protected by somatic-cell “bodies” to colonize the higher-lethality (> 50%) sector of the environment. In general, long-lived but only moderately-protective (β = 0.5) somatic cells enable small colonies at moderate lethality (Figure 5(b)), while shorter-lived but fully-protective (β = 0) somatic cells enable larger colonies even at high lethality (Figure 5(c)). Combining these somatic-cell advantages enables invasions of the entire environment (Figure 5(d)). The increasing occupation of the high-lethality (*b* > 0.5) part of the environment as the protection of stem cells by somatic cells increases is shown in Figure 6.

In these simulations, stem cells that are fully enclosed by a somatic “body” cannot escape to generate new progeny. They can and do, however, explore the external environment whenever one of the surrounding somatic cells dies; most such pioneering stem cells die before reproducing if the environmental lethality is greater than 50%. “Budding” of a stem cell with its surrounding somatic cells from a large connected mass to form an independent colony has been observed in some simulation runs.

### Percolation theory describes a phase transition to informational opacity

To characterize the phase transition to multicellularity more precisely, consider the environment **E** as a source and the cell **C** as a receiver of VFE, i.e. prediction-violating information. We can represent protective cells and/or their internal processes collectively as a channel that probabilistically decreases the transmitted VFE, i.e. has an effective β < 1. Any such channel can, alternatively, be decomposed into a network of smaller channels, each of which transmits VFE with a well-defined probability, with interconnections between these channels representing cross-modulation or other probabilistic dependency. Such a network constitutes a “Markov blanket” as defined by Pearl [47] around the cell **C** (technically, the internal states of cells directly interacting with both **E** and **C** form the Markov blanket; “expanding” each cell into a network of internal processes creates a multi-layer Markov blanket). Markov blankets provide a general model of the interactions between biological systems and their environments [3,10]. We assume here that the conditional dependencies that define the Markov blanket around the cell **C** are in a state of flux and can be affected by regulatory changes within **C** (in the language of active inference, actions by **C**) that alter either its sensitivity to environmental VFE or the behavior and hence protective capabilities of its neighboring cells. Decreasing exposure to environmental VFE is then a matter of **C** finding the right set of conditional dependencies, i.e. the right Markov blanket, to interpose between itself and **E**.

**Figure 7.**
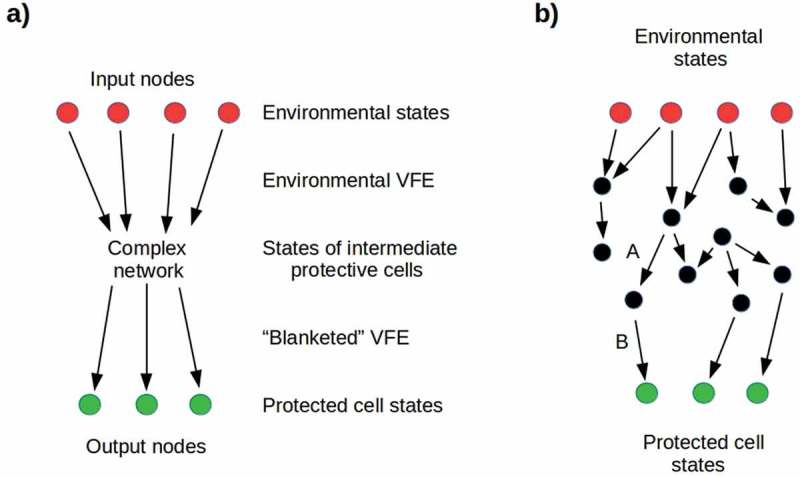
a) Simplified representation of a Markov blanket. Input nodes represent environmental states that transmit environmental VFE to a network of intermediate nodes, with conditional dependencies specified by a connectivity matrix M_*ij*_. Output nodes represent protected cell states that receive the “blanketed” VFE. It is natural to interpret distinct input nodes as transmitting the VFE of different dimensions of the environment, e.g. pathogenicity or toxicity. b) A network very near its percolation threshold: removing either link A or B results in opacity. On the other hand, adding any downward-going link between black nodes, or between black and green nodes, increases the cell’s exposure to its environment.

If we only consider transmission of VFE from **E** to **C**, ignoring the corresponding flow from **C** to **E** (i.e. the reaction map ***R***) as above, we can represent the Markov blanket as a weighted directed graph with nodes {*k*} and connection weights given by a real matrix M with elements M_*ij*_ representing the probability of transmitting VFE from node *i* to node *j*. The M_*ij*_ represent, in this case, the coupling or conditional dependency between the states *i* and *j*. Some subset {*k*_*in*_} of the nodes represent states of **E**, while another, disjoint subset {*k_out_*} of nodes represent states of **C** as shown in Figure 7; intermediate nodes represent states of intermediate cells, e.g. non-reproductive somatic cells surrounding **C**. In the simplest case, transmission of VFE through the blanket can be considered effective only for values of the conditional dependencies M_*ij*_ above some threshold; hence these can be considered binary without loss of generality. The effective transmission from **E** to **C** is, in this case, given by counting paths between them. A binary blanket in which every pair of nodes is connected has the maximal number of paths; one in which no nodes are connected has no paths. If {*k*_*in*_} ∪ {*k_out_*} = {*k*} (i.e. the graph representing the Markov blanket is bipartite), only if M_*ij*_ = 0 for all *i, j* are there no paths from **E** to **C**; otherwise this is not the case.

Markov blankets in which every input node is connected by a path to some output node and vice-versa fully expose **C** to **E**, i.e. correspond to β = 1. Such blankets are in a natural sense “transparent” to VFE. States in which no path exists from any input node to any output node fully protect **C** from **E**, correspond to β = 0, and are in a natural sense “opaque.” The transition from only local communication between nodes (i.e. opacity) to global communication between essentially all nodes (i.e. transparency) as connections are randomly added to an information-transmission network has been extensively studied using percolation theory, e.g. in models of epidemics [48,49], predator-prey interactions [50], intracellular coordination of mitochondrial function [51], and long-range signaling in microbial communities [52]. Such transitions are characterized by a critical point, the percolation threshold, at which opacity switches exponentially to transparency or, as in the problem of interest here, transparency switches exponentially to opacity. The number of connections that must be added to reach the percolation threshold from below, or removed to reach it from above, depends strongly on the size, local connectivity, and density of long-range connections in the starting graph [48]. In particular, the percolation threshold rapidly decreases as the number of long-range connections in the starting graph is increased.

If we consider the input nodes of the Markov blanket around a cell to correspond to environmental states characterized by distinct dimensions of environmental lethality, e.g. pathogenicity or toxicity, long-range connections in the blanket correspond to multiple paths by which the cell can be exposed to each dimension of lethality. The relatively low percolation threshold of such a blanket means that many connections have to be severed to induce opacity and hence protection. A blanket in which each input node is linked to only one output node has fewer long-range connections, a higher percolation threshold, and hence a smaller number of links that must be severed to induce opacity (Figure 7(b)). One would, therefore, expect successful cells to produce protective progeny that are each specialized to deal with one kind of environmental threat, and to regulate the morphology and/or metabolism of each of these cell types to maximize its opacity, i.e. to minimize the exposure to environmental VFE of the protected cell by decreasing the VFE transmission of its Markov blanket. The tendency of regulatory [53], metabolic [54], and cell-cell communication [55] networks toward small-world [56] structure, in which long-range connections link distant clusters of nodes, renders this kind of specialization difficult to achieve.

### The ancestral genetic toolkit provides signals for controlling daughter cells

Successful construction of a protective Markov blanket out of daughter cells requires two genetic capabilities: the ability to arrest the daughter’s cell cycle and the ability to modulate its gene expression and metabolism away from independent motility and continued cell division and toward adhesion, a specialized morphology, and the production of specialized products. Both comparative genomics and the cell and developmental biology of unicellular eukaryotes increasingly show that these capabilities were available in the genetic toolkit of ancestral holozoans.

As noted earlier, prespore cells in migratory *Dictyostelium* secrete morphogens (Differentiation-Inducing Factors or DIFs) that arrest the cell cycle and induce terminal differentiation in prestalk cells [37,38]. Native *Dictyostelium* DIFs have been shown to deactivate the Wnt pathway and suppress Cyclin D1 and hence cell division in mammalian cells by activating the GSK-3β kinase [57]. While the Wnt pathway appears to be unique to metazoans [58], GSK-3, a β-catenin homolog, and Frizzled homologs are present in *Dictyostelium* and are involved in cAMP-induced migration and cell adhesion [59] and epithelial polarity [60]. Additional signals involved in *Dictyostelium* spore differentiation, including the metazoan neurotransmitters GABA and glutamate, have also been identified and in part characterized [61]. Hence it is reasonable to assume that morphogens capable of both inducing cell-cycle arrest and regulating motility and adhesion are available to eukaryotes at least since the last common ancestor of Amoebozoa and Opisthokonts.

The availability of both whole-genome and sample sequences from choanoflagellates and earlier-branching holozoans confirms that not all, but many metazoan cell polarity, adhesion, signaling, and transcriptional control systems predate the choanoflagellate – metazoan split and may be ancestral to holozoans in general [1,62], with adhesion and motility regulators such as Src [63], the Rac/Rho second messengers [64], and cell-division regulators such as Myc [65] as prominent examples. The ancient roots of hormonal [66,67] and neurotransmitter [68,69] systems suggest that both were available to reproductive cells as a means of modulating or controlling the behavior of non-reproductive progeny in the earliest obligate multicellular organisms.

## Discussion

### Somatic cells as information-processing modules

In all metazoans, distinct differentiated cell types perform distinct functions. From the perspective adopted here, these distinct functions can be seen as information-processing functions. Distinct non-reproductive cell types act in different ways to reduce environmental VFE, and to replace it, in their interactions with the reproductive cells with which they share inclusive fitness, with highly-predictable protective behavior. The modularization of protective and other supportive behaviors in distinct cell types is consistent with the vast expansion and diversification of transcription factors, relative to the unicellular eukaryotic toolkit, seen in multicellular eukaryotic lineages (reviewed by [70]). Such systematic reduction of VFE is precisely the role ascribed to functional networks of the nervous system by Bayesian approaches to cognition [9,71,72]. This view of somatic cells in general as essentially cognitive [73–75] suggests an integrative, scale-free approach to biological information processing that spans all of phylogeny, in which the sensory, regulatory and behavioral roles of the mammalian nervous system are seen as fundamentally analogous to the sensory, regulatory and behavioral roles of metabolic processes in unicellular organisms [76].

Within an “imperial” model of multicellularity that views reproductive, i.e. stem or germ, cells as both regulatory controllers of and receivers of information from surrounding somatic tissue [24], the VFE reduction performed by somatic cells is in service of the genetic interests of their parent stem/germ cells, interests which they share via inclusive fitness. Reproductive fitness remains, in this model, at the level of stem/germ cells; it is not necessary to define an independent “organismal” level of reproductive fitness as it is in aggregation followed by cooperation models [7,8]. Re-assertion of individual reproductive fitness by previously somatic cells (i.e. cancer) is not “cheating” on a cooperative “contract” that divides cellular labor [20] but rather escape from regulatory control. Somatic mutation is tolerated in this “imperial” setting provided it does not reprogram somatic cells toward division, i.e. cancer.

### Cancer as an escape from “imperial” oversight

As noted earlier, re-allocation of resources away from reproduction is, on this model, one of the key enablers of multicellularity. Without such re-allocation, cells within an aggregate compete both for resources and genetically; the latter form of competition has been recently demonstrated in cytokinesis-inhibited *Saccharomyces cerevisiae* aggregates [77]. Somatic cells that reprogram to either normal reproductive cells or cancer cells must re-allocate resources to cell division, including high-turnover production of nucleotide bases, membrane components, and proteins [78–80]. Such reprogramming is inducible by multiple signals in vertebrates, the receptors or effectors for many of which (e.g. Src, Rho and Myc as noted earlier) are present in the ancestral toolkit.

By reprogramming metabolism for division, cancer cells are re-asserting an independent genetic interest and abandoning their previous role as somatic protectors of “normal” reproductive stem, non-stem progenitor or, in obligate sexual species, germ cells [20,81]. From their perspective, the host body is a protective environment which they can exploit to their own ends. One would expect, therefore, cancer cells to transmit regulatory signals to surrounding tissue, whether somatic or reproductive, to suppress cell division and otherwise minimize detectable VFE. Such suppression by tumor cells of cell division in surrounding non-tumor somatic tissue has been observed [82,83].

### Neural signaling as a mechanism for enforcing somatic cell fates

The nervous system is a multicellular eukaryotic innovation, evident as a network of cell-cell communication in Porifera [84], supported by specialized cells in Ctenophores, and fully developed in Cnidarians and Bilaterians [85,86]. Nervous systems are traditionally thought of as providing sensing and behavioral coordination functions at the level of the whole organism. The current model suggests an additional, deeply ancestral function of the nervous system: distributed control of cell proliferation. As bodies become more complex geometrically, local suppression of somatic-cell proliferation by individual stem cells becomes insufficient to maintain a regular shape; hence sponges, with interstitial stem cells but no long-distance neurons, are amorphous, but all other metazoans, with neurons, are not. The current model predicts that neural communication is directly involved in the regulation of somatic cell proliferation, and hence in the control of morphology. While innervation is known to be important for regeneration [87] and the nascent CNS has recently been found to be crucial for the patterning of distant somatic regions [88], the role of innervation in regulating proliferation, apoptosis, and neoplasia remains an active area for future research (reviewed by [89,90]).

Obligate sexuality with a differentiated germline places a second demand on proliferation control: germ cells must assure that non-germ stem cells do not proliferate indefinitely [23,24]. As germline cells are typically segregated early in development while stem cells remain relatively dispersed, regulation of stem cell proliferation requires a long-range mechanism. In organisms like *C. elegans* with invariant cell lineages [91], the solution is, in effect, for the germline cells to be the only adult stem cells. The current model predicts that in organisms with adult stem cells, stem cell proliferation will be under regulatory control by the germ line, with neural and/or hormonal systems as effectors.

## Conclusions

The selection pressures that drove the transitions to obligate multicellularity with complex morphology in plant and animal lineages remain poorly understood. Here we have suggested that multicellularity solves a problem for proliferating cells: the problem of predicting the behavior of the environment. By surrounding themselves with reproductively-disabled copies of themselves, proliferating cells, which then become stem cells, provide for themselves a local environment that buffers the VFE of the larger environment outside. On this model, the transition to obligate multicellularity can be viewed as a phase transition in which the blanket of surrounding somatic cells becomes effective opaque to environmental variation. Maintaining this opacity is, as Clark [92] has pointed out, the key to maintaining autopoesis [93] for the blanketed system as a whole. As only a satisficing solution is required, the opacity of the blanket is not expected to be complete in any but the most challenging environments.

This model of the transition to multicellularity is consistent with an “imperial” view of the multicellular state as one in which stem cells effectively control the differentiation and behavior of somatic cells. It suggests that the fundamental role of neural signaling is the control of cell fate, and that cancer is an escape by somatic cells from the non-proliferating fate imposed on them by stem cells.

## Notes

1. For a given organism, the VFE of the environment is its complexity (the Kullback-Leibler (KL) divergence between posterior and prior probability distributions) minus the organism’s predictive accuracy. The KL divergence measures the effective dimensionality of the environment, i.e. its dimensionality as it is represented by the organism. Representations (generative models [9,10]) with high dimensionality may be prone to overfitting and hence brittleness; reducing the effective dimensionality by coarse-graining provides a solution to this problem, albeit one that may miss significant details.

2. As VFE depends on the KL divergence between posterior and prior probability distributions, changing either distribution separately changes the VFE. Here we refer to any change in the posterior distribution made by the organism as an “action” on the environment and refer to any change in the prior probability distribution made by the organism as a “regulatory” change. The latter correspond to alterations of the organism’s generative model of the environment, e.g. by evolution or learning.

## Declarations

### Availability of data and material

Simulation model code is available at https://chrisfieldsresearch.com/somatic-protection-model.htm; non-commercial re-use permitted with citation.

## Abbreviations

Mya, Million years ago; FEP, Free-Energy Principle; VFE, Variational free energy
